# Identification of early mild cognitive impairment using multi-modal data and graph convolutional networks

**DOI:** 10.1186/s12859-020-3437-6

**Published:** 2020-11-18

**Authors:** Jin Liu, Guanxin Tan, Wei Lan, Jianxin Wang

**Affiliations:** 1 Hunan Provincial Key Lab on Bioinformatics, School of Computer Science and Engineering, Central South University, 932 Lushan South Road, Changsha, 410083 China; 2 School of Computer, Electronics and Information, Guangxi University, 100 Daxue East Road, Nanning, 530004 China

**Keywords:** Early mild cognitive impairment, Multi-modal MRI data, Graph convolutional networks, Identification

## Abstract

**Background:**

The identification of early mild cognitive impairment (EMCI), which is an early stage of Alzheimer’s disease (AD) and is associated with brain structural and functional changes, is still a challenging task. Recent studies show great promises for improving the performance of EMCI identification by combining multiple structural and functional features, such as grey matter volume and shortest path length. However, extracting which features and how to combine multiple features to improve the performance of EMCI identification have always been a challenging problem. To address this problem, in this study we propose a new EMCI identification framework using multi-modal data and graph convolutional networks (GCNs). Firstly, we extract grey matter volume and shortest path length of each brain region based on automated anatomical labeling (AAL) atlas as feature representation from T1w MRI and rs-fMRI data of each subject, respectively. Then, in order to obtain features that are more helpful in identifying EMCI, a common multi-task feature selection method is applied. Afterwards, we construct a non-fully labelled subject graph using imaging and non-imaging phenotypic measures of each subject. Finally, a GCN model is adopted to perform the EMCI identification task.

**Results:**

Our proposed EMCI identification method is evaluated on 210 subjects, including 105 subjects with EMCI and 105 normal controls (NCs), with both T1w MRI and rs-fMRI data from the Alzheimer’s Disease Neuroimaging Initiative (ADNI) database. Experimental results show that our proposed framework achieves an accuracy of 84.1% and an area under the receiver operating characteristic (ROC) curve (AUC) of 0.856 for EMCI/NC classification. In addition, by comparison, the accuracy and AUC values of our proposed framework are better than those of some existing methods in EMCI identification.

**Conclusion:**

Our proposed EMCI identification framework is effective and promising for automatic diagnosis of EMCI in clinical practice.

## Background

Alzheimer’s disease (AD) is a common neurodegenerative disease accompanied by memory, cognitive and motor disorders. As of 2006, there are 26.6 million AD patients worldwide, and by 2050, one in every 85 people worldwide is expected to have AD [1]. As the world is developing into an aging society, the negative impact of AD on families and society will become more and more significant. Mild cognitive impairment (MCI) is an intermediate process in the conversion of normal people to AD, with up to 15% of people with MCI being converted to AD each year [2]. At present, there is no accurate diagnosis and effective treatment for AD. Most researchers hope that patients can be diagnosed in time when they are in the stage of MCI, and then take effective measures to prevent further deterioration of the disease. Therefore, accurate identification of early MCI (EMCI) is critical to human health.

Since magnetic resonance imaging (MRI) can noninvasively measure brain structural and functional changes related to brain disorder development *in* *vivo*, in recent years it has been widely used in the study of brain disorders [3], such as AD/MCI [4, 5], schizophrenia [6, 7] and autism [8]. Therefore, MRI can provide phenotypes that can be used to diagnose such disorders. MRI falls into two broad categories: structural MRI (such as T1 MRI, and T2 MRI) and functional MRI (such as rs-fMRI and ts-fMRI). Brain structure is typically measured using structural MRI, which can provide relatively high-definition brain structure in grey matter and white matter. There are many metrics to measure brain structure, and most of them have been widely applied in the study of MCI identification, such as grey matter volume, cortical thickness, texture properties and so on [9–12]. Brain function is typically measured using functional MRI, which can provide changes in hemodynamics caused by neuronal activity. Functional connectivity between brain regions is a common measure of brain function. Also, brain networks based on brain regions and functional connectivity between brain regions have been widely used for feature representation in the study of various brain disorders. In the past years, brain function analysis based on graph theory has shown a powerful role in exploring functional impairment of brain disorders, and has been widely used for MCI identification [13–16].

In the past decade, whether structural MRI-based brain structure metrics or functional MRI-based brain function metrics, these metrics were mainly used separately in the studies with MCI. For example, Karas et al. [9] found that the MCI subjects showed a decrease in grey matter volume in the medial temporal lobe. Wang et al. [15] constructed functional brain networks of MCI subjects and found that the length of the shortest path increased in MCI subjects compared with NCs; Zhang et al. [17] first extracted functional connectivity between brain regions from functional MRI data of each subject as feature representation, and then trained a L2-regularized logistic regression classifier based on these functional connectivity features to perform MCI identification. Therefore, many researchers believe that different metrics may contain different-yet-complementary information, and combinations of these metrics may improve MCI classification performance over separate metrics. In fact, recent studies have also been show great promises for improving the accuracy of MCI identification by combining multiple structural and functional metrics, such as grey matter volume (GMV) and shortest path length (SPL). For example, Wee et al. [18] first used both structural MRI and functional MRI data of each subject to construct multiple brain networks for each subject, and then extracted local clustering coefficient from each brain network of each subject as feature representation to perform the MCI identification task by using a multi-kernel learning algorithm; De Marco et al. [19] used multiple machine learning models based on different metrics from both structural MRI and functional MRI data to investigate the performance of MCI identification; Tripathi et al. [20] proposed an unsupervised framework for the classification of EMCI and LMCI by combining shape and voxel-based features from 12 brain regions; Jie et al. [21] proposed a feature combination framework to combine both temporal and spatial features of dynamic functional networks to perform automatic identification of EMCI and LMCI. So far, although some results have been achieved for the identification of MCI subjects based on structural and functional MRI data, extracting which features and how to combine multiple features to improve MCI identification accuracy have always been a difficult problem.

Recently, deep learning models have been widely applied in the fields of medical health [22–25]. Since spectral graph-based convolutional neural network (GCN) models [26, 27] can process irregular graph structures using computational harmonic analysis, many researchers in medical health adopt spectral GCN models to perform various applications, especially at a subject level [28–32]. For example, Anirudh et al. [28] proposed a bootstrapping strategy-based spectral GCN model to perform autism spectrum disorder classification using rs-fMRI data. Guo et al. [29] proposed a spectral GCN model that integrates brain connectivity information to predict visual tasks using MEG data. Ktena et al. [30] proposed a siamese GCN model to learn a graph similarity metric to perform autism and sex classification using rs-fMRI data. In the field of MCI identification, the spectral GCN model is also applied. For example, Parisot et al. [31] proposed a spectral GCN model by combining imaging and non-imaging information to distinguish EMCI from late MCI. However, Parisot et al. [31] only extract GMV of each brain region as imaging features from T1w MRI data, and the Mini Mental State Examination (MMSE), a common scale for AD in clinical practice, has not been taken into account.

**Fig. 1:**
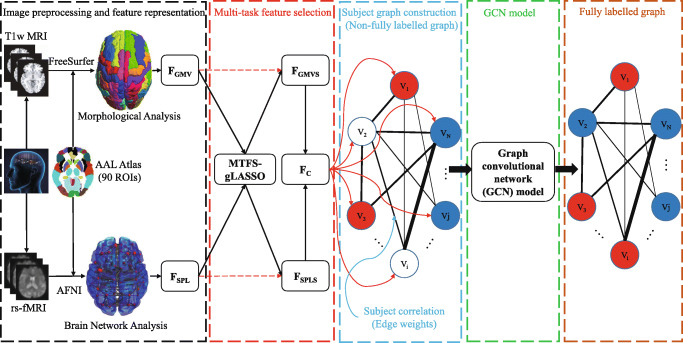
Schematic overview of our proposed EMCI identification framework (GCN-EMCI)

Taking the above-mentioned into consideration, in this study we propose a new EMCI identification framework using multi-modal data and graph convolutional networks, which is denoted as GCN-EMCI and shown in Fig. 1. Firstly, we extract GMV and SPL of each brain region based on automated anatomical labeling (AAL) atlas [33] as feature representation from T1w MRI and rs-fMRI data of each subject, respectively. Then, in order to obtain features that are more helpful in identifying EMCI, a common multi-task feature selection method is applied. Afterwards, we construct a non-fully labelled subject graph using imaging and non-imaging phenotypic measures of each subject. Finally, a recent GCN model is adopted to perform the EMCI identification task. The GCN-EMCI is evaluated on 210 subjects (including 105 subjects with EMCI and 105 NCs) with T1w MRI and rs-fMRI data from the Alzheimer’s Disease Neuroimaging Initiative (ADNI) database (http://adni.loni.usc.edu/).

## Materials and methods

### Data

A subset of the Alzheimer’s Disease Neuroimaging Initiative (ADNI) [34] is used to evaluate our proposed EMCI identification method. This subset includes 210 subjects with both T1w MRI and rs-fMRI data, which are composed of 105 normal controls (NCs) and 105 subjects with early mild cognitive impairment (EMCI). All T1w MRI and rs-fMRI data are acquired on 3.0 Tesla Philips medical system scanners at multiple sites, and the slice thickness of T1w MRI data and rs-fMRI data is 1.2 mm and 3.0 mm, respectively. Furthermore, rs-fMRI data of each subject contain 140 volumes. Table 1 presents a brief demographic information of the subjects involved in this study. In Table 1, the front and back of ± represent mean and standard deviation, respectively. For more details with these subjects from ADNI, please see http://adni.loni.usc.edu/.

**Table 1:** Demographic information of the subjects involved in this study

Demographic information	NC	EMCI	*p*-value
Number (male/female)	105 (54/51)	105 (49/56)	>0.05
Age (year)	77.1±6.3	76.3±5.4	>0.05
MMSE	29.1±1.1	27.5±1.8	>0.05

Prior to EMCI identification using the subjects, Chi-square test [35] is implemented to test the differences in gender, and *t*-test [35] is implemented to test the differences in age and MMSE. As can be seen from Table 1, no significant differences (*p*>0.05) are found between each of the two groups in gender, age and MMSE.

### Image preprocessing and feature representation

As can be seen from Fig. 1, the procedures of image preprocessing and feature representation in our work mainly include two aspects: T1w MRI data preprocessing and feature representation, and rs-fMRI data preprocessing and feature representation. These two aspects are briefly introduced as follows.

**Fig. 2:**
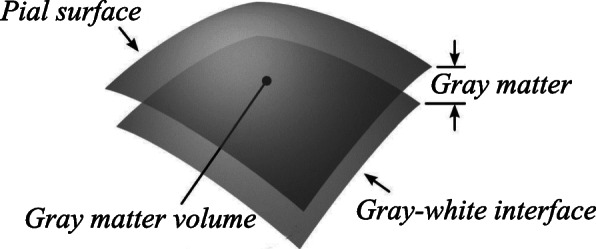
A sketch of calculating the gray matter volume

Firstly, a standard preprocessing procedure is applied to T1w MRI data of each subject using a standard FreeSurfer pipeline (https://surfer.nmr.mgh.harvard.edu) [36], including motion correction, non-uniform intensity normalization, talairach transform computation, skull removal, volumetric segmentation, cortical surface reconstruction and so on. After this standard preprocessing procedure, we can obtain the gray matter (GM) map, which lies between the gray-white interface and the pial surface, and has been widely used to investigate AD/MCI in the literatures [37, 38]. Since EMCI is accompanied by brain atrophy, we suspect that the gray matter volume (GMV) is also accompanied by a decrease. The gray matter volume is defined as the amount of gray matter, and uses the surface-based volume calculation as shown in Fig. 2. For this reason, in this study we extract GMV based on each GM region of the automated anatomical labeling (AAL) atlas [33] as structural feature representation from T1w MRI data for each subject. For more details of the 90 GM regions of the AAL atlas, please see http://www.gin.cnrs.fr/en/tools/aal-aal2/. Finally, we can obtain GWV of each GM region from T1w MRI data for each subject, which is denoted as *F*_*GMV*_. It is worth mentioning that *F*_*GMV*_ is 90-dimensional vectors.

Secondly, a standard preprocessing procedure is also applied to rs-fMRI data of each subject using the pipeline provided by the Analysis of Functional NeuroImages (AFNI) software (https://afni.nimh.nih.gov/) [39], including removing the first 10 rs-fMRI volumes, slice timing, head motion corrections, spatial smoothing, band-pass filtering (0.01-0.1 Hz), nuisance signal regression, and Montreal Neurological Institute (MNI) space normalization and so on. After this standard preprocessing procedure, we can obtain the average rs-fMRI time series of each brain region according to the AAL atlas from the resulted rs-fMRI data of each subject. At present, brain network analysis based on graph theory [40, 41] plays an important role in the study of complex brain diseases, and is widely used in various brain diseases [42], such as AD/MCI, Schizophrenia, Parkinson and so on. The shortest path length (SPL) plays an important role in the information transmission of a brain network, and it is a very important metric to describe the internal structure of the brain network. Meanwhile, the SPL is a measure of functional integration, which can measure the ability to rapidly combine pieces of specialized information. The shorter SPL can transmit the information more quickly and reduce brain consumption. Since EMCI is accompanied by brain atrophy, we suspect that the brain information transmission of EMCI is also accompanied by damage. For this reason, in this study we construct an individual brain network for each subject, which consists of 90 brain regions according to the AAL atlas and functional connectivity between each two brain regions. The functional connectivity is calculated by the pairwise Pearson correlation coefficient between the average rs-fMRI time series of each two brain regions. Then, we compute nodal SPL based on individual brain network of each subject as functional feature representation, which is denoted as *F*_*SPL*_. It is worth mentioning that *F*_*SPL*_ is also 90-dimensional vectors.

With the above analysis, we can obtain two regional feature sets from both T1w MRI and rs-fMRI data for each subject, i.e., *F*_*GMV*_ and *F*_*SPL*_. These two regional feature sets are taken as the original feature representation of each subject.

### Multi-task feature selection

Since the two original feature sets: *F*_*GMV*_ and *F*_*SPL*_ are calculated based on a certain rule, these two feature sets may contain irrelevant or redundant features. Therefore, feature selection is required for these two original feature sets. Before performing feature selection on each feature set, each original feature set were first scaled individually to range [−1,+1]. Then, every scaled feature was normalized across all training subjects to obtain its standard score (z-value). These steps ensure that each feature set is within the same scale, minimizing possible bias that may occur when performing selection on features with different dynamic ranges.

In this study, to consider the relationship between different feature selection tasks, the different feature selection tasks should be learned jointly, which is often called multi-task feature selection (MTFS). At present, the group LASSO-based MTFS method (denoted as MTFS-gLASSO) [43] is a common feature selection method, and has been widely used in various feature selection tasks. The MTFS-gLASSO method can be formulated as follows,

1 $$ \mathop {\min }\limits_{\boldsymbol{W}} \left({\sum\limits_{t = 1}^{T} {\left\| {{\boldsymbol{y}} - {\boldsymbol{X}_{t}}{\boldsymbol{w}_{t}}} \right\|_{2}^{2}} + \lambda {{\left\| \boldsymbol{W} \right\|}_{2,1}}} \right)  $$

where ${\boldsymbol {X}_{t}} = \left [ {\boldsymbol {x}_{t}^{1},\boldsymbol {x}_{t}^{2},...,\boldsymbol {x}_{t}^{i},...,\boldsymbol {x}_{t}^{N}} \right ]^{'} \in {\Re ^{N \times P}}$ denotes all training subjects in the *t*-th task, *P* denotes the number of features of each training subject, $\phantom {\dot {i}\!}\boldsymbol {y}=[y^{1},y^{2},...,y^{i},...,y^{N}]^{'} \in {\Re ^{N}}$ denotes the labels of all training subjects, $\boldsymbol {W} = \left [ {{\boldsymbol {w}_{1}},{\boldsymbol {w}_{2}},...,\boldsymbol {w}_{i},...,{\boldsymbol {w}_{T}}} \right ] \in {\Re ^{P \times T}}$ is a discriminant matrix, ∥***W***∥_2,1_ denotes the *l*_2,1_−norm of ***W***, and *λ*>0 is a parameter to balance the loss function (i.e., $\sum \limits _{t = 1}^{T} {\left \| {{\boldsymbol {y}} - {\boldsymbol {X}_{t}}{\boldsymbol {w}_{t}}} \right \|_{2}^{2}}$) and the regularization term (i.e., ∥***W***∥_2,1_). The larger the *λ* value, the greater the penalty for the parameters in the model, resulting in higher model sparsity, that is, more parameters are trained to zero.

Finally, as shown in Fig. 1, we concatenate the two selected feature sets (i.e., *F*_*GMVS*_ and *F*_*SPLS*_) obtained by MTFS-gLASSO, which is denoted as *F*_*C*_.

### Subject graph construction

Before performing EMCI identification using GCN model, we should fist construct a graph using all subjects. A graph is typically defined as *G*=(*V*,*E*,*C*), where *V* is the set of vertices (or nodes), *E* is the set of edges, and *C* is the adjacency matrix describing the graph’s connectivity. Therefore, to construct the subject graph, we need to determine the definition of the nodes and edges in this graph.

In this study, we define each subject as a node, and the correlation between each two subjects as edges. For a node *V*_*i*_, we use *F*_*C*_(*V*_*i*_) to represent it. To compute the adjacency matrix (i.e., *C*) of the subject graph, we follow the work of Parisot et al. [31]. Considering a set of *D* non-imaging phenotypic measures *H*={*H*_*d*_} (such as gender, age or MMSE), the adjacent matrix is defined as follows:

2 $$ C\left({i,j} \right) = Corr\left({V_{i},V_{j}} \right)\sum\limits_{d = 1}^{D} {\eta \left({{H_{d}}\left(V_{i} \right),{H_{d}}\left(V_{j} \right)} \right)}  $$

3 $$Corr\left({V_{i},V_{j}} \right) = \exp \left({ - \frac{{{{\left[ {\ell \left({F_{C}\left(V_{i} \right),F_{C}\left(V_{j} \right)} \right)} \right]}^{2}}}}{{2{\sigma^{2}}}}} \right)  $$

4 $$ \eta \left({{H_{d}}\left(V_{i} \right),{H_{d}}\left(V_{j} \right)} \right) = \left\{ \begin{array}{l} 1{\quad \mathrm{ if }}\left| {{H_{d}}\left(V_{i} \right) - {H_{d}}\left(V_{j} \right)} \right| < \varepsilon \\ 0{\quad \mathrm{ otherwise}} \end{array} \right.  $$

where *Corr*(*V*_*i*_,*V*_*j*_) is an imaging phenotypic measure of correlation between subjects, *ρ*(·,·) is a correlation distance, *σ*>0 is a constant parameter, and *η*(*H*_*d*_(*V*_*i*_),*H*_*d*_(*V*_*j*_)) a non-imaging phenotypic measure of distance between subjects. It is worth mentioning that different non-imaging phenotypic measures correspond to different values of *ε*.

### Classification using GCN model

**Fig. 3:**
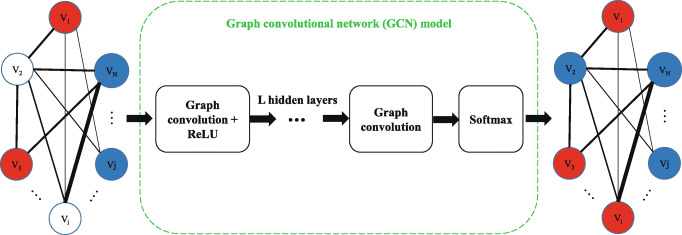
Schematic illustration of the GCN model in GCN-EMCI

Following the work of Parisot et al. [31], schematic illustration of the GCN model in this study is shown in Fig. 3. The input layer of the GCN model is a non-fully labelled subject graph, and the output layer of the GCN model is a fully labelled subject graph. Obviously, the training set consists of labelled nodes (such as *V*_1_ and *V*_*j*_) in the non-fully labelled subject graph, and the testing set consists of unlabelled nodes (such as *V*_2_ and *V*_*i*_) in the non-fully labelled subject graph.

As can be seen from Fig. 3, the GCN model is a semi-supervised classification method and a relatively simple model, which consists of L hidden layers with graph convolution and a softmax layer. The L hidden layers are activated by a rectified linear unit (ReLU) function. After training the GCN model, the softmax function is used in the testing set to assign labels to nodes that have no labels.

## Experiments and results

### Experimental settings

Our proposed EMCI identification framework (GCN-EMCI) is evaluated on 105 subjects with EMCI and 105 NCs via a 5-fold cross-validation strategy. In the multi-task feature selection step, the parameter *λ* is set to [0, 100] with a step size of 5, and these features with non-zero elements in ***W*** are selected. In the subject graph construction step, the corresponding *ε* values for the non-imaging phenotypic measures: gender, age and MMSE are set to 1, 2 and 2, respectively. In the classification using GCN model step, GCN parameters are similar with [31], we only change: L = 5, dropout rate: 0.01, learning rate: 0.02, epochs: 500, K = 4.

To quantitatively evaluate the classification performance of GCN-EMCI, in this study the three metrics: accuracy (ACC), sensitivity (SEN) and specificity (SPE) are computed. In addition, to quantitatively evaluate the overall performance of GCN-EMCI, the area under receiver operating characteristic (ROC) curve (AUC) value [44–46] is also reported. It is worth mentioning that the greater the values of the four metrics are, the better the classification performance of the method is.

To avoid the bias caused by randomly dividing the subjects in the cross-validation, the 5-fold cross-validation is repeated 50 times in our experiments. In this study we only report the average of 50 repeated experiments.

### Classification performance

In order to verify the effectiveness of GCN-EMCI, we have done a series of experiments based on different subject graphs. First, we only use imaging phenotypic features (i.e., *F*_*GMV*_, *F*_*SPL*_, *F*_*GMV*_+*F*_*SPL*_, *F*_*GMVS*_, *F*_*SPLS*_ and *F*_*C*_) to construct subject graphs, and then we combine imaging and non-imaging phenotypic features (i.e., *F*_*C*_+*H*) to construct subject graph. The results of these experiments are shown in Table 2.

**Table 2:** Classification performance of GCN-EMCI based on different subject graphs

Features	ACC(%)	SEN(%)	SPE(%)	AUC
*F*_*GMV*_	65.8	69.8	62.7	0.672
*F*_*SPL*_	62.7	66.5	59.4	0.637
*F*_*GMV*_+*F*_*SPL*_	69.7	71.4	65.6	0.719
*F*_*GMVS*_	79.8	83.4	77.1	0.802
*F*_*SPLS*_	75.3	78.3	73.2	0.765
*F*_*C*_	81.5	82.7	80.2	0.828
*F*_*C*_+*H*	84.1	86.5	81.3	0.856

As can be seen from Table 2, the classification performance of GCN-EMCI based on subject graphs with original features (i.e., *F*_*GMV*_, *F*_*SPL*_ and *F*_*GMV*_+*F*_*SPL*_) are relatively low (such as ACC <70%), and the classification performance of GCN-EMCI based on subject graphs with selected features (i.e., *F*_*GMVS*_, *F*_*SPLS*_ and *F*_*C*_) are relatively good (such as ACC ≈ 80%). However, the classification performance of GCN-EMCI based on subject graph with both imaging features and non-imaging features (i.e., *F*_*C*_+*H*) is the best (ACC = 84.1%, SEN = 86.5%, SPE = 81.3%, AUC = 0.856). Experimental results show that GCN-EMCI is effective for EMCI identification.

## Discussion

### Different feature selection methods

To demonstrate the superiority of MTFS-gLASSO in GCN-EMCI, we compare two other common feature selection methods, i.e., *t*-test [35] and LASSO [47]. These two methods are implemented by scipy [48] and scikit-learn [49] packages in Python, respectively. The specific approach of these two methods is to first use the t-test or LASSO method for *F*_*GMV*_ and *F*_*SPL*_ respectively, and then concatenate *F*_*GMVS*_ and *F*_*SPLS*_ as the feature representation of each subject. It’s worth mentioned that when t-test is used as feature selection method, these features with *p*-value less than 0.1 are selected; when LASSO is used as feature selection method, these features whose weights are not equal to 0 are selected. The EMCI/NC classification performance based on GCN-EMCI with different feature selection methods is shown in Table 3.

**Table 3:** Comparison with different feature selection methods for EMCI/NC classification

Methods	ACC(%)	SEN(%)	SPE(%)	AUC
t-test	70.9	74.7	68.2	0.728
LASSO	78.5	83.6	76.6	0.798
MTFS-gLASSO	84.1	86.5	81.3	0.856

As can be seen from Table 3, the EMCI/NC classification performance based on GCN-EMCI with MTFS-gLASSO is best in ACC, SEN, SPE and AUC. This result indicates that MTFS-gLASSO can obtain better feature representation than the other two feature selection methods.

### Comparison with existing methods

To demonstrate the superiority of GCN-EMCI, we also compare two existing methods [20, 21] in EMCI identification. In our comparative experiments, the existing methods are also repeated 50 times via a 5-fold cross-validation strategy, and the average classification performance is reported in Table 4. In order to statistically verify that the classification performance of GCN-EMCI is better than that of the other two existing methods, we also report the *p*-values of GCN-EMCI and other methods in terms of ACC, which is shown in Table 4.

**Table 4:** Comparison with existing methods for EMCI/NC classification

Methods	ACC(%)	SEN(%)	SPE(%)	AUC	*p*-value
Tripathi et al., 2017 [20]	75.8	74.2	76.7	0.762	<0.01
Jie et al., 2018 [21]	79.5	82.6	77.2	0.801	<0.01
GCN-EMCI	84.1	86.5	81.3	0.856	

As can be seen from Table 4, GCN-EMCI obtains the best performance in ACC, SEN, SPE and AUC for EMCI/NC classification. Compared with the two existing methods, GCN-EMCI utilizes the correlation between each two subjects, and uses a GCN model to learn the deep differences between EMCI and NC. In addition, as the statistical *p*-value is less than 0.01, GCN-EMCI is significantly better than the other two existing methods. These results indicate that our proposed method (i.e., GCN-EMCI) is not only effective, but also has a good advantage in EMCI identification.

## Conclusion

In this study, we propose a new EMCI identification method using multi-modal data and graph convolutional networks. Firstly, we perform image preprocessing and feature representation for both T1w MRI and rs-fMRI data of each subject. Then, in order to obtain features that are more helpful in identifying EMCI, a common multi-task feature selection method is adopted. Afterwards, we construct a subject graph using imaging phenotypic measures and non-imaging phenotypic measures of each subject. Finally, a GCN model is applied to perform the EMCI identification task. Experimental results on 210 subjects from ADNI database demonstrate that our proposed framework is effective for EMCI identification. This method paves the way to discriminative imaging markers for computer-aided identification of EMCI.

## Data Availability

All data from the Alzheimer’s Disease Neuroimaging Initiative (ADNI) are available to qualified researchers via the ADNI Access Management System (http://adni.loni.usc.edu/data-samples/access-data/). The datasets used and/or analysed during the current study are available from the corresponding author on reasonable request.

## Abbreviations

AAL: automated anatomical labeling
ACC: accuracy
AD: Alzheimer’s disease
ADNI: Alzheimer’s Disease Neuroimaging Initiative
AFNI: Analysis of Functional NeuroImages
AUC: area under ROC curve
EMCI: early mild cognitive impairment
GCN: graph convolutional network
GMV: grey matter volume
LASSO: least absolute shrinkage and selection operator
LMCI: late mild cognitive impairment
MCI: mild cognitive impairment
MEG: magnetoencephalography
MMSE: Mini Mental State Examination
MNI: Montreal Neurological Institute
MRI: Magnetic Resonance Imaging
MTFS-gLASSO: LASSO-based MTFS
MTFS: multi-task feature selection
NC: normal control
ReLU: rectified linear unit
ROC: the receiver operating characteristic
rs-fMRI: resting state-functional MRI
SEN: sensitivity
SPE: specificity
SPL: shortest path length
ts-fMRI: tasking state-functional MRI
